# No superior method for analgesia after total knee arthroplasty: randomised controlled comparison of adductor canal block combined with iPACK block versus posterior capsule block

**DOI:** 10.1007/s00402-025-05845-5

**Published:** 2025-04-05

**Authors:** Mehmet Fevzi Cakmak, Serkan Bayram, Levent Horoz, Fatma Nur Arslan, Onur Utku Demir, Safa Gürsoy

**Affiliations:** 1 Department of Orthopedics and Traumatology, Kirsehir Ahi Evran University, Kirsehir, Turkey; 2 Department of Orthopedics and Traumatology, Istanbul Faculty of Medicine, Istanbul University, Istanbul, Turkey; 3 Department of Anesthesiology, Kirsehir Ahi Evran University, Kirsehir, Turkey; 4 Department of Orthopedics and Traumatology, Acibadem University, Istanbul, Türkiye; 5 Istanbul Faculty of Medicine, Department of Orthopedics and Traumatology, Istanbul University, Çapa Fatih Istanbul, Istanbul, 34093 Turkey

**Keywords:** Total knee arthroplasty, Adductor canal blockage, iPACK blockage, Visual analog scale

## Abstract

**Objective:**

The aim of this study compare the effectiveness of the space between the popliteal artery and the posterior knee capsule (iPACK) and posterior capsule injection (PCI) in patients with primary end stage knee osteoarthritis treated with total knee arthroplasty (TKA).

**Methods:**

This was a double-blind, prospective, randomised trial. A total of 195 participants were randomly assigned to one of three groups: Group 1 with an adductor canal block (ACB) plus iPACK. Group 2 with ACB + PCI and a final control group with ACB only. All participants underwent primary total knee arthroplasty. Outcome measures comprised pain assessment using the Visual Analog Scale (VAS) and monitoring opioid analgesic consumption. VAS measurements were taken at the 1st, 6th, 12th, 24th, 48th, and 72nd hours, followed by the 10th day and the 12th week.

**Results:**

Age, sex, BMI and side of surgery were analyzed and no significant differences were found. Groups ACB + iPACK and ACB + PCI exhibited significantly lower VAS scores compared to the control group at 3, 6, and 12 h after surgery, with group ACB + iPACK showing the lowest VAS scores among all groups. No significant difference in VAS values between groups was detected after 24 h postoperatively and after that. Significant differences were observed between groups in opioid consumption. The values for the first hour, first day, second day, and total consumption exhibited statistically significant differences between the groups.

**Conclusion:**

Our study has shown that PCI in combination with ACB is not inferior to the iPACK technique. It is our belief that these combination techniques can be used in accordance with the surgeon’s experience and preference. It is important to remember that PCI is quicker and easier to perform without using ultrasonography.

## Introduction

After a successful total knee arthroplasty (TKA), full recovery is expected within 3–6 months, and the patient returns to life without pain [1, 2]. Pain control is a prerequisite for the initiation of early rehabilitation and the achievement of favorable clinical outcomes in TKA patients. Surgical techniques and implant technology for TKA have advanced significantly in recent years. Despite these advances, approximately 20% of TKA patients report pain, reduced physical function and inadequate postoperative recovery [3]. The reasons are many and include a wide range of factors, both knee-related and unrelated [4, 5].

The anatomical complexity of the area, which is innervated from many sites, is the main reason for the difficulty in managing pain after TKA. Depending on the location of potential pain, different blocks may need to be used [6, 7]. Blockade of the saphenous branch of the femoral nerve within the adductor canal is effective in the prevention of anterior knee pain [8, 9]. Numerous methods have been developed to control pain in the posterior part of the knee, which is innervated by the obturator, sciatic, and tibial nerves. Direct blocks to these nerves are effective in pain control but may result in unpredictable losses of motor function [10, 11]. Hence, different methods have been described for analgesia in this area. Perioperative periarticular infiltration (PAI) and posterior capsule infiltration (PCI), along with postoperative interspace between the popliteal artery and capsule of the posterior knee (iPACK), are among these methods [12, 13].

In this prospective randomized study, we aimed to compare the effectiveness of iPACK and PCI in patients with primary end stage knee osteoarthritis treated with TKA.

## Materials and methods

This trial was approved by the Institutional Review Board and registered in the Clinical Trials Registry as NCT05943080. The trial was conducted in accordance with the tenets of the current version of the Declaration of Helsinki. Participants were asked to volunteer to participate. Participants were informed that their personal information would remain confidential, and their consent was obtained.

### Sample size calculation

The analyses were conducted using the G. Power-3.1.9.7 software. Based on the power analysis, the total sample size for the F-test, with a 0.05 error rate and 95% confidence interval, is 159, with a 0.20 error rate and 80% power. Due to the possibility of using non-parametric tests in the analyses, it is recommended to increase the sample size by 15% (Lehmann, 2006) [14]. In our study, we determined that the number of samples should be 195 (65 patients for per group) consider the patients who might drop out of the study.

### Study population

**Fig. 1:**
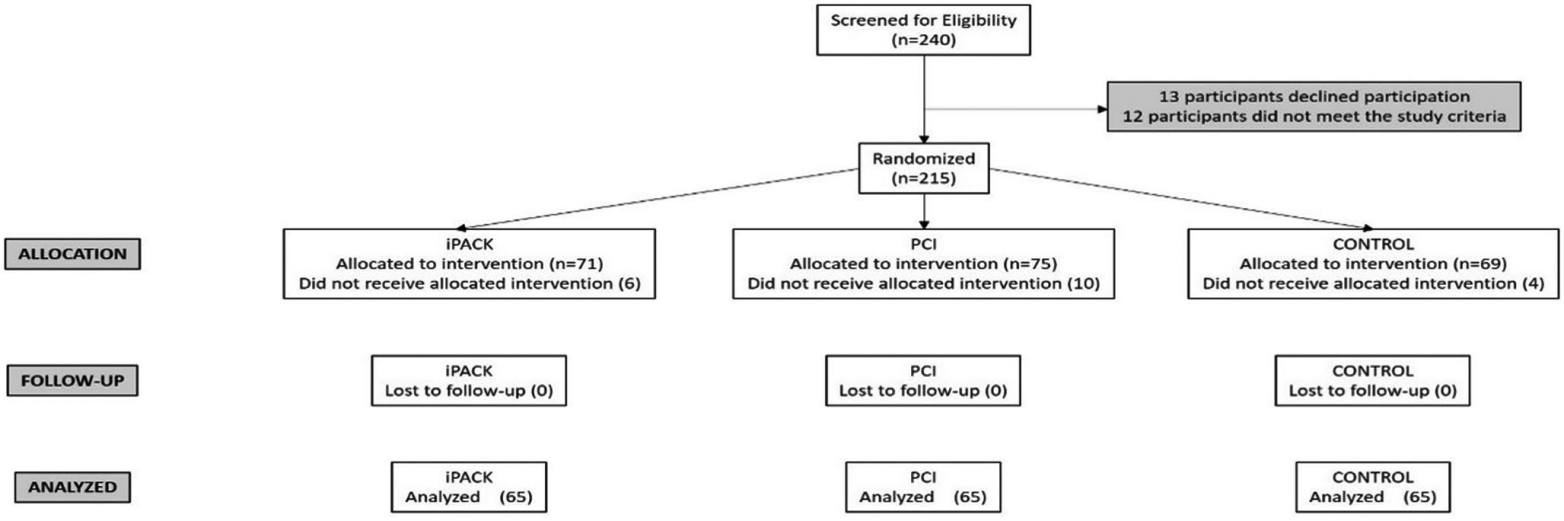
CONSORT Diagram

Patients with end stage knee osteoarthritis, underwent primary knee arthroplasty and to agree to take part in the study were included this study. *Patients* declined participation in the study, allergy to analgesics or contraindications to anesthesia, *a*dministration of narcotic pain relievers prior to surgery, *n*europathy, *c*irrhosis and renal failure (creatinine > 1.2) were excluded. 240 participants underwent TKA due to end-stage (Kellgren-Lawrence grade 4 knee osteoarthritis between the date. Thirteen individuals declined participation, and it has been determined that 12 individuals did not meet the study criteria. Furthermore, due to factors such as changes in surgical or anesthesia techniques, 20 participants were excluded from the study. The final count of participants stands at 195. Each group consisted of 65 participants. The CONSORT diagram is illustrated in the Fig. 1.

### Study design and participants

This study has been designed in a double-blind, randomized, controlled trial format. Surgery began in April 2023 and was completed in December 2023 for patients enrolled in the study. However, due to the completion of follow-up data, the trial was registered between July 2023 and March 2024. The participants were randomly allocated into three groups, one of which was the control group. Both the participants and surgeons were blinded regarding group assignments.

All participants underwent primary total knee arthroplasty with the tourniquet and cement. A surgeon with 10 years of experience in the field of total knee arthroplasty (M.F.C.) performed all total knee arthroplasty procedures.

### Adductor Canal block (ACB) procedure

**Fig. 2:**
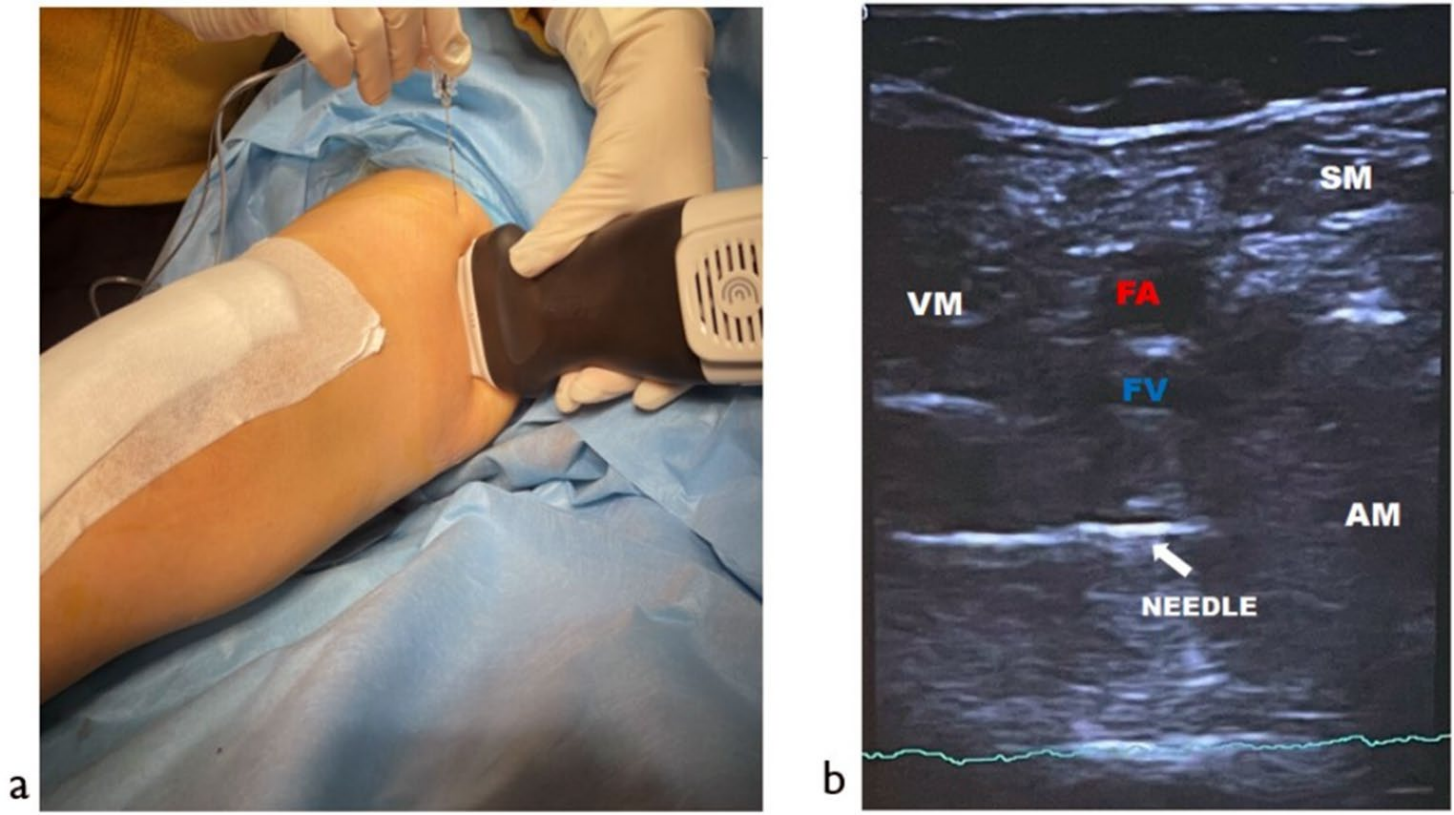
**a**) Postoperative ultrasonographic visualization adductor blockage with sterile condition. **b**) FA - Femoral Artery, FV - Femoral Vein, VM - Vastus Medialis Muscle, S - Sartorius Muscle, AM - Adductor Magnus Muscle

All of the adductor canal blocks were performed under ultrasound guidance by the same anesthetist, who had more than five years of experience. The patient’s leg was first turned slightly outwards in the supine position. The femoral artery was visualized with the ultrasound probe from the midline of the thigh, and the level where the femoral artery coincided with the midline instead of the sartorius muscle was selected for the block. Then, a 100 mm block needle was inserted at a 45-degree angle, 4–5 cm lateral to the probe, and the adductor sheath was entered lateral to the adductor artery. After it was seen that there was no aspirated blood in the drug line of the needle, 15 ml of 0.25% bupivacaine (7 ml of 0.5% bupivacaine and 8 ml of serum physiologic mixture) was sent into the adductor canal (Fig. 2).

### PCI procedure

**Fig. 3:**
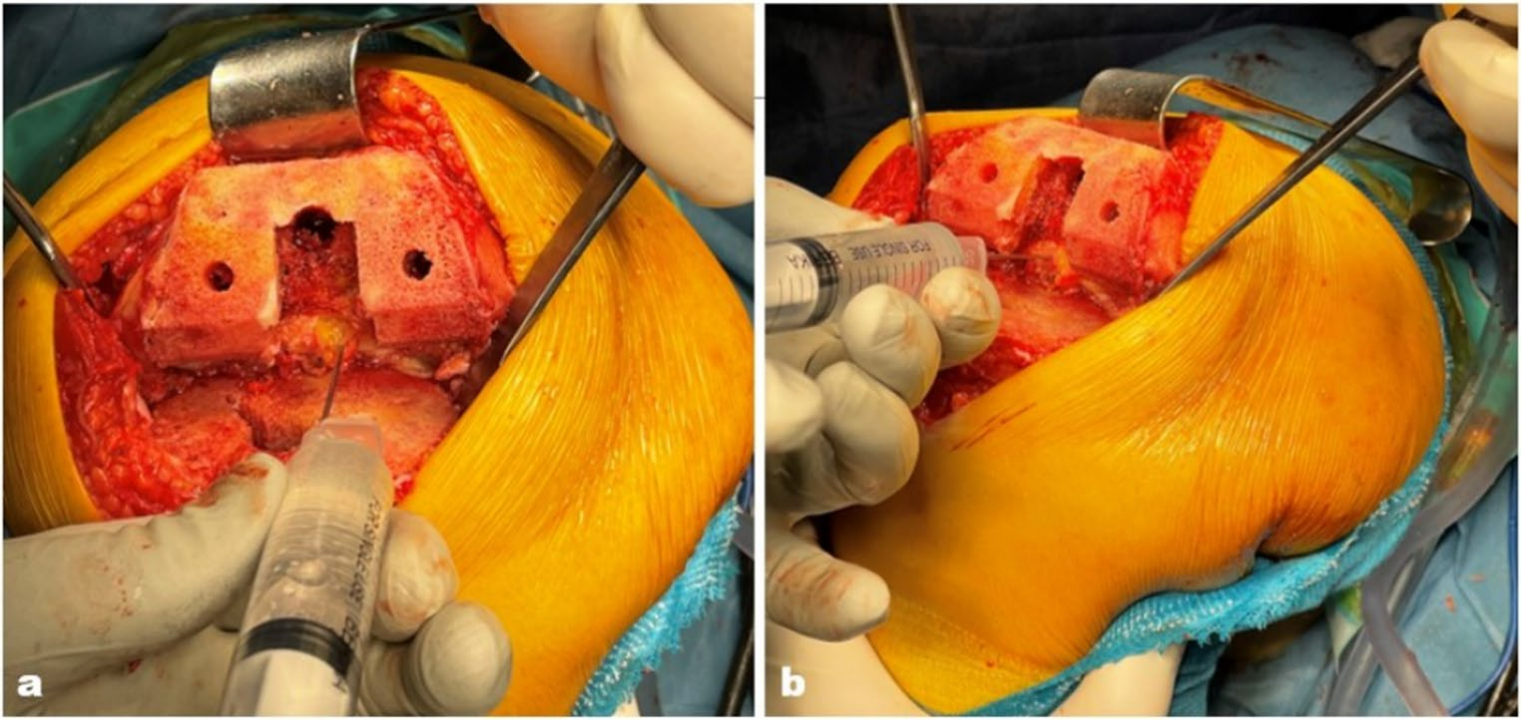
PCI is performed after the femoral and tibial cutting are made

The PCI procedure was performed after the irrigation procedures and prior to implantation. The procedure protocol entailed the injection of a 20 mL PCI cocktail (0.2% ropivacaine + 2.0 mg/mL epinephrine) into the posterior capsule through the intercondylar space of the femur. In the posterior femoral intercondylar space, the capsule has been penetrated at medial and lateral points. It has been ascertained that the injector is not within a vascular structure, following which infiltration has been performed (Fig. 3).

### iPACK procedure

**Fig. 4:**
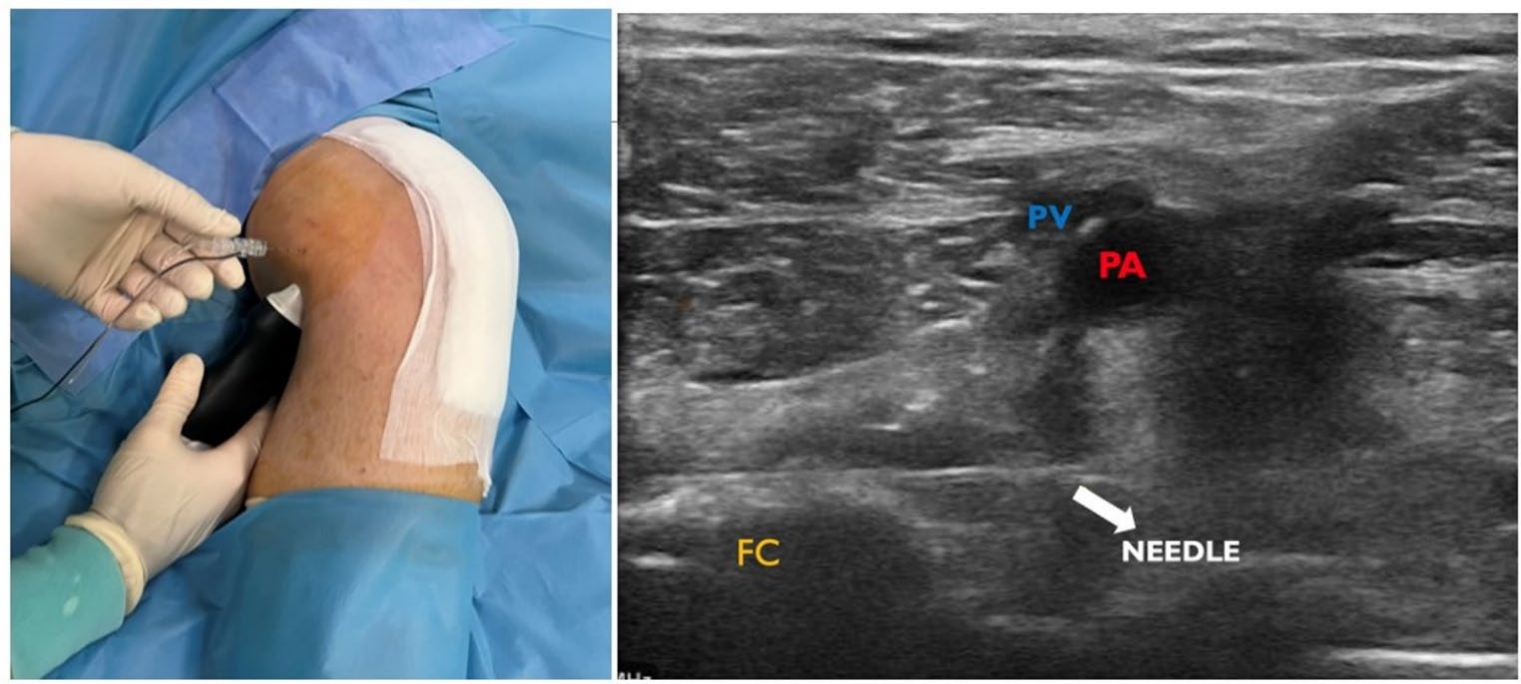
The anatomical landmarks for the iPACK procedure are identified. Clivage is achieved by blunt dissection of the finger 7 cm proximal to the joint line. A block is performed by entering at an angle of 45 degrees

For the iPACK block performed by the anesthetist under ultrasonography guidance, the patient’s leg in the supine position was lifted, turned into external rotation at the hip joint, and slightly flexed at the knee joint. The ultrasound probe is placed below the knee to visualize the medial and lateral condyles and the popliteal artery. Then, the probe was directed proximally, and the level at which the condyles disappeared was selected for the block. A 100 mm block needle was advanced from the medial knee at a 90° angle, 3–4 cm above the probe. The needle was visualized and advanced between the femur and popliteal artery until the end of the soft tissue on the lateral side of the knee. Again, after the drug line was aspirated and it was seen that there was no blood, 20 mL of the cocktail (consisting of 0.2% ropivacaine and 2.0 mg/mL epinephrine) was injected under imaging guidance by withdrawing the needle and confirming the tissue distribution (Fig. 4). The iPACK procedure was carried out in the post-anaesthesia care unit in the immediate post-operative period. This period of time corresponds to an average of 10 min after the operation.

- Group 1: ACB + iPACK. The procedures were performed post-operatively with the assistance of ultrasound by an anesthesiology specialist.
- Group 2: ACB + PCI. The ACB procedure was performed post-operatively with the assistance of ultrasound by an anesthesiology specialist. Anatomical landmarks will be identified, and the surgeon will execute the PCI procedure intraoperatively without additional imaging.
- Group 3: This is the control group. Only ACB was performed post-operatively with the assistance of ultrasound by an anesthesiology specialist.

### Pain management

Postoperative pain management included metamizole sodium (500 mg every 6 h), celecoxib (oral, once/day, 400 mg), paracetamol (oral, once/day, 500 mg), and pregabalin (oral, once/day). 75 mg per dose). An ampoule (2 ml) of 100 mg tramadol hydrochloride was given as rescue therapy if the pain persisted and the patient could not tolerate it.

VAS and the consumption of opioids are the primary outcomes. ROM, OKS, KSS (clinical and functional), and continuous passive motion (CPM) values are seconder outcomes.

Age, gender, body mass index (BMI), and side of operation. The participants’ need and consumption of opioids. (One ampoule (2 ml) contains 100 mg of tramadol hydrochloride). Active Range of Motion (ROM) of the knee joint. Measured prior to surgery, on the 3rd and 10th days, and at the 12th week post-operation.

Parameters for rehabilitation are termed as the Knee Society Score-clinic (KSS-c), Knee Society Score-functional (KSS-f), and the Oxford Knee Score (OKS). Evaluated before surgery and again at the 12th week post-operation.

Visual Analog Scale (VAS) assessments were conducted pre-surgery. Post-surgery measurements were taken at the 1st, 6th, 12th, 24th, 48th, and 72nd hours, followed by the 10th day and the 12th week.

Continuous physical therapy was implemented, utilizing a Continuous Passive Motion device to gauge the patient’s endurance at specific movement degrees. Post-surgery, the device was initially set to 45° to commence rehabilitation. Patients who tolerated a 60-minute session progressed gradually to 75°, 90°, 105°, and finally 120°. On the first day, the maximum angle achieved was 90°. On the second day, those tolerating it progressed to 105° and 120°. The tolerated angles and durations for the 1st, second, and third days were recorded, along with the participants’ passive maximum joint flexion degrees and endurance level.

### Statistical analysis

In the statistical analyses, the SPSS 26 software was utilized. The conformity of variables to the normal distribution was examined using the Kolmogorov-Smirnov test. The Kruskal-Wallis test was employed to compare three independent groups, while the Wilcoxon Signed-Rank test was used for two related measurements and the Friedman test for three or more repeated measures. In instances where significant differences were identified in the results of the Kruskal-Wallis and Friedman tests, Bonferroni correction was applied to ascertain the source of significance. Relationships between categorical variables were investigated using the Chi-Square test. In these analyses, a p-value of less than 0.05 was considered statistically significant.

## Results

The age, gender, BMI values, and the side on which the procedure was performed were analyzed across the groups. No significant differences were observed in these parameters among groups. (Table 1).

**Table 1:** The age, BMI and the side values of groups

		iPACK + ACB Mean ± SD *n* (%)	PCI + ACB Mean ± SD *n* (%)	Control (ACB) Mean ± SD *n* (%)	*p* value
Age		66,22 ± 6,845	65,49 ± 9,033	67,2 ± 7,641	0,431^*^
BMI		33,79 ± 6,283	33,41 ± 3,752	34,73 ± 6,026	0,606^*^
Side	Left	33 (50,8)	31 (47,7)	34 (52,3)	0,866^**^
	Right	32 (49,2)	34 (52,3)	31 (47,7)	

^*^Kruskal Wallis ^**^Chi-Square

Analysis of functional scores showed that the mean VAS score at hour 3 was 4.7 ± 1.2 in the iPACK + ACB group, 5 ± 1.07 in the PCI + ACB group and 7.06 ± 1.4 in the control (isolated ACB) group. The iPACK + ACB and PCI + ACB groups showed a significantly greater improvement in pain scores compared to the control (isolated ACB) group (*p* < 0.001 and *p* < 0.001 respectively). Although the iPACK + ACB group showed a slightly greater improvement in VAS scores than the PCI + ACB group, the difference was not significant (*p* > 0.05). The mean VAS score at hour 6 was 5,4 ± 1,4 in the iPACK + ACB group, 5,83 ± 0,8 in the PCI + ACB group and 6,8 ± 1,6 in the control (isolated ACB) group. The iPACK + ACB and PCI + ACB groups showed a significantly greater improvement in pain scores compared to the control (isolated ACB) group (*p* < 0.001 and *p* < 0.001 respectively). Although the iPACK + ACB group showed a slightly greater improvement in VAS scores than the PCI + ACB group, the difference was not significant (*p* > 0.05). Similarly, the mean VAS score at hour 12 was 4,4 ± 1,2 in the iPACK + ACB group, 5,02 ± 1 in the PCI + ACB group and 6,1 ± 1,8 in the control (isolated ACB) group. The iPACK + ACB and PCI + ACB groups showed a significantly greater improvement in pain scores compared to the control (isolated ACB) group (*p* < 0.001 and *p* < 0.001 respectively). Although the iPACK + ACB group showed a slightly greater improvement in VAS scores than the PCI + ACB group, the difference was not significant (*p* > 0.05). (Table 2). No significant difference in VAS values between groups was detected after 24 h postoperatively and after that.

**Table 2:** Comparison of VAS score between the groups

	İPACK + ACB Mean ± SD	PCI + ACB Mean ± SD	Control (ACB) Mean ± SD	*p* value	Difference
VAS Preoperative	8,65 ± 0,959	8,86 ± 1,074	8,48 ± 1,032	0,069	-
VAS postop 3 h	4,72 ± 1,206	5,00 ± 1,075	7,06 ± 1,402	**0**,**000**	1 < 3;2 < 3
VAS POSTOP 6 h	5,42 ± 1,413	5,83 ± 0,821	6,80 ± 1,603	**0**,**000**	1 < 3;2 < 3
VAS POSTOP 12 h	4,42 ± 1,211	5,02 ± 1,023	6,11 ± 1,847	**0**,**000**	1 < 3;2 < 3
VAS POSTOP 24 h	4,29 ± 1,100	4,42 ± 0,900	4,43 ± 1,287	0,703	-
VAS POSTOP 2 day	4,25 ± 1,687	4,49 ± 1,161	4,62 ± 1,711	0,237	-
VAS POSTOP 3 day	3,42 ± 1,540	3,69 ± 1,014	3,75 ± 1,649	0,093	-
VAS POSTOP 10 day	2,85 ± 1,395	3,34 ± 1,350	3,31 ± 1,530	0,082	-
VAS POSTOP 12 week	1,71 ± 0,843	1,63 ± 0,993	1,71 ± 0,824	0,651	-

The first hour of opioid requirement was 8,6 ± 1,1 in the iPACK + ACB group, 8,2 ± 3,9 in the PCI + ACB group and 3,8 ± 1,9 in the control (isolated ACB) group. The control group (isolated ACB) had a significantly earlier need for opioids than iPACK + ACB and PCI + ACB group (*p* < 0.001 and *p* < 0.001 respectively). The mean opioid consumption of first day was 1,31 ± 0,49 in the iPACK + ACB group, 1,3 ± 0,48 in the PCI + ACB group and 1,7 ± 0,5 in the control (isolated ACB) group. The iPACK + ACB and PCI + ACB groups showed a significantly lower opioid consumption compared to the control (isolated ACB) group (*p* < 0.001 and *p* < 0.001 respectively). The mean opioid consumption of second day was 1,29 ± 0,55 in the iPACK + ACB group, 1,26 ± 0,56 in the PCI + ACB group and 1,6 ± 0,58 in the control (isolated ACB) group. The iPACK + ACB and PCI + ACB groups showed a significantly lower opioid consumption compared to the control (isolated ACB) group (*p* < 0.001 and *p* < 0.001 respectively). The iPACK + ACB group had lower opioid consumption than the PCI + ACB group. However, the difference was not significant (*p* > 0.05) (Table 3).

**Table 3:** Comparison of opioid consumption between the groups

	iPACK + ACB Mean ± SD	PCI + ACB Mean ± SD	Control (ACB) Mean ± SD	*p*	Difference
First hour of opioid requirement	8,62 ± 1,128	8,23 ± 3,932	3,88 ± 1,916	**0**,**000**	3 < 1;3 < 2
Opioid consumption of first day	1,31 ± 0,498	1,35 ± 0,482	1,77 ± 0,553	**0**,**000**	1 < 3;2 < 3
Opioid consumption of second day	1,29 ± 0,551	1,26 ± 0,567	1,60 ± 0,581	**0**,**000**	1 < 3;2 < 3
Opioid consumption of third day	0,98 ± 0,515	0,92 ± 0,714	0,97 ± 0,612	0,647	-
Total opioid consumption	3,60 ± 0,981	3,54 ± 1,359	4,34 ± 1,241	**0**,**000**	1 < 3;2 < 3

The values related to the KSS-c, KSS-f, and OKS variables were analyzed for differences among the groups. It was determined that there were no significant differences between the groups (*p* > 0.05). (Table 4). The findings of the difference analysis conducted between groups regarding active ROM, CPM motion, and endurance values are presented in Table 5. Following the analyses, it was determined that the values of ROM on the third post-op day, CPM 1 motion, CPM 2 motion, CPM 2 endurance, and CPM 3 endurance exhibited significant differences between the groups. However, no significant differences were found in the values of the other variables (Table 5).

**Table 4:** Comparison of functional outcome between the groups

	i PACK + ACB Mean ± SD	PCI + ACB Mean ± SD	Control (ACB) Mean ± SD	*p*
KSS Knee Pre-op	38,92 ± 10,295	40,29 ± 4,749	39,26 ± 13,404	0,06
KSS Knee Post-op 12 Week	88,05 ± 4,299	89,72 ± 5,644	88,23 ± 4,499	0,09
KSS FONK Pre-op	44,08 ± 13,460	44,20 ± 15,171	42,18 ± 12,932	0,60
KSS FONK Post-op 12 Week	88,62 ± 4,801	88,57 ± 8,391	88,00 ± 4,984	0,54
OKS Pre-op	12,02 ± 3,843	12,17 ± 4,878	12,62 ± 4,516	0,66
OKS Post-op 12 Week	40,35 ± 3,233	39,15 ± 4,128	40,11 ± 3,628	0,13

SD: standard deviation; KSS: Knee society score; OKS: Oxford Knee Score

**Table 5:** Comparison of clinical outcomes between the groups

	iPACK + ACB Mean ± SD	PCI + ACB Mean ± SD	Control (ACB) Mean ± SD	*p*	Difference
ROM Pre-operative	95,69 ± 8,965	96,23 ± 6,314	97,08 ± 9,181	0,759	-
ROM Post-op 3 Day	90,54 ± 7,022	95,69 ± 9,917	89,62 ± 5,883	**0**,**000**	1 < 2;3 < 2
ROM Post-op 10 Day	96,62 ± 8,529	97,77 ± 9,015	97,62 ± 8,344	0,576	-
ROM Post-op 12 Week	111,77 ± 6,756	112,85 ± 7,014	112,38 ± 7,077	0,789	-
CPM1 Motion	51,00 ± 17,549	52,38 ± 14,819	48,69 ± 9,197	**0**,**003**	3 < 2
CPM1 Endurance	77,31 ± 16,180	81,85 ± 19,275	79,08 ± 9,678	**0**,**000**	1 < 2;3 < 2
CPM2 Motion	56,31 ± 7,969	54,69 ± 12,433	53,92 ± 9,078	0,145	-
CPM2 Endurance	91,31 ± 6,572	94,69 ± 18,369	90,54 ± 7,506	**0**,**000**	1 < 2;3 < 2
CPM3 Motion	56,15 ± 7,845	56,31 ± 11,868	56,08 ± 9,272	0,322	-
CPM3 Endurance	101,62 ± 7,960	101,46 ± 18,951	99,38 ± 10,136	**0**,**001**	1 < 2;3 < 2

SD: Standard deviation; ROM: Range of motion; CPM: continuous passive motion

## Discussion

The most significant finding(s) of the study were the VAS scores in the first 12 h. These were significantly lower in the ACB+İPACK and ACB + PCI groups compared to the control group. The difference in VAS scores reached the level of clinical significance (more than 1 score difference) at 3 h, 6 h and 12 h postoperatively in comparison to the control group. However, the difference between the iPACK group and the PCI group did not reach the level of clinical significance. Interspace between the popliteal artery and capsule of the posterior knee and PCI are known to be effective in postoperative pain management [13, 15, 16]. However, the iPACK technique’s targeted approach is indisputably more specific compared to periarticular applications. Cadaver studies examining iPACK have shown that the dye spreads along the popliteal fossa without involving the proximal sciatic nerve, staining nerves that innervate the posterior capsule [17]. The PCI technique aims for the space between the popliteal artery and the capsule through just two points of entry from the posterior intercondylar space rather than multiple points of local infiltration [18–20]. Existing literature suggests that ACB combined with iPACK and PAI is superior to isolated ACB [21–24]. Mou et al. have shown that the combination of iPACK + ACB is superior to either İPACK or ACB alone [13]. It is not surprising that the combined use of these techniques, which are known to control pain in different areas of the knee, produces better results. Similarly, Sankineani et al. found the ACB+İPACK combination more successful than isolated ACB. However, the absence of a control group in their study renders the results open to question [23]. In the study by Zhou et al., the combination of ACB with PCI was compared with ACB plus local infiltration anesthesia. They used isolated ACB as a control group in their research. Their findings support the use of ACB + PCI as the ideal post-operative analgesic protocol for the first 24 h. Our study confirms their findings and supports this protocol by demonstrating the efficacy of the ACB + PCI application [22].

In their prospective study, Pai and colleagues compared iPACK + ACB with PAI + ACB, examining 92 patients (24). They found no significant difference in VAS scores, opioid consumption, and functional outcomes. Although similar in design to our study, they investigated PAI, whereas we focused on PCI. Additionally, our study’s robustness is enhanced by a higher participant count and the use of a control group, which elevates the level of evidence and reliability of the results. Kertkiatkachorn and colleagues, in their randomized controlled study of 76 patients, compared ACB + iPACK, PAI, and ACB + PAI [25]. They demonstrated that the ACB + iPACK combination provided similar levels of analgesia as the ACB + PAI combination but without statistical superiority. However, it was noted that participants in the ACB + iPACK group required higher amounts of opioids and had poorer functional performance. This data is not congruent with our study. In our research, both the iPACK and PCI groups were found to be analogous in terms of both opioid consumption and functional outcomes.

A meta-analysis examined randomized prospective research that compared isolated single-shot ACB applications with combined iPACK applications. The study reported that the combined approach was superior in terms of pain scores and opioid consumption. Our research also found that combined applications were superior to ACB alone. However, no significant differences in clinical scores were reported between groups in the meta-analysis and our study [24].

A review study investigated isolated and combined PAI, iPACK, and peripheral nerve block applications [26]. Our findings support the conclusion that the best analgesic effect is achieved by combining PAI, iPACK, and peripheral nerve blocks, facilitating satisfactory pain control and preservation of motor functions [13, 23, 27, 28]. Clinical outcomes in the first 24 h after surgery are similar for the analgesic techniques compared in many studies [13, 20]. However, a number of studies have shown that combined applications give better results in terms of early range of motion (ROM) which is supported by our study [23, 24, 29]. Unlike VAS and opioid usage, on the third postoperative day, the active ROM value of the ACB + PCI group and the first three days’ passive motion and endurance values measured with CPM were better compared to other groups. This could be explained by the better pain scores of the combination groups than the ACB group. However, the reason why the ACB + PCI group outperformed the ACB + iPACK group might be, as Kertkiatkachorn and colleagues mentioned, due to the lower ambulatory ability in patients with iPACK. Although these slight differences are statistically significant, their clinical manifestation is challenging to observe and diminishes by the 10th postoperative day. Despite similar clinical outcomes, the presence of early pain should be noticed in the etiology of chronic pain [5]. A major concern in our study was neurovascular injury during perioperative PCI or intravenous solution administration. However, there was no evidence of such complications in any of the participants in our study.

Our study possesses certain limitations. Primarily, a comparison of different block techniques applied for analgesia in various knee regions was not conducted, with pain scoring segregated by areas. This is attributed to the anticipated difficulties in participant compliance with such a procedure. Another limitation is the absence of allergy testing for local anesthetics. The rationale for not conducting this test lies in asserting local anesthetic safety in high-participant-number studies [29]. The reliance on participant compliance for using the VAS also constitutes a limitation of our study.

## Conclusion

The interspace between the popliteal artery and the posterior knee capsule is a reliable analgesia technique that is effective in reducing pain during the first 12 h and significantly reduces opioid consumption. However, our study has shown that PCI in combination with ACB is not inferior to the iPACK technique. It is our belief that these combination techniques can be used in accordance with the surgeon’s experience and preference. It is important to remember that PCI is quicker and easier to perform without using USG.
